# MTHFD1L-associated branched-chain amino acid degradation transcriptional signature represents a conserved metabolic program in Epstein-Barr virus-associated malignancies

**DOI:** 10.3389/fcimb.2026.1955009

**Published:** 2026-10-01

**Authors:** Jianmei Zhou, Hu Hao, Wenxia Zhang, Lin Mao, Xiaoran Zhao, Jie Zhu

**Affiliations:** 1 Department of Gastroenterology, Taihe Hospital, Hubei University of Medicine, Shiyan, China; 2 School of Clinical Medicine, Shandong Second Medical University, Weifang, China; 3 Department of Gastroenterology, The First Affiliated Hospital of Shandong First Medical University & Shandong Provincial Qianfoshan Hospital, Jinan, China

**Keywords:** branched-chain amino acid degradation, EBV-associated malignancies, Epstein–Barr virus, metabolic reprogramming, MTHFD1L, single-cell RNA sequencing

## Abstract

Epstein-Barr virus (EBV)-associated malignancies encompass multiple tumor types with distinct tissue origins, yet the extent to which they share conserved metabolic programs remains poorly understood. Here, we performed an integrated cross-cancer transcriptomic analysis of nasopharyngeal carcinoma, EBV-associated gastric cancer, and EBV-associated plasmablastic lymphoma to identify conserved metabolic programs, followed by independent cohort validation and single-cell transcriptomic characterization. We identified 103 consensus differentially expressed genes and 72 conserved gene–metabolic pathway pairs across the three discovery cohorts. Among them, only the MTHFD1L—associated Valine, leucine and isoleucine degradation program was consistently preserved in both the discovery and validation cohorts. Single-cell analysis further demonstrated that this conserved metabolic program was predominantly enriched in CD4^+^ T cells, γδ T cells, and NK cells, and was accompanied by cell state-dependent metabolic remodeling, altered cell–cell communication, and distinct MTHFD1L-centered regulatory networks across immune cell populations. Collectively, our study identifies an MTHFD1L-associated branched-chain amino acid degradation transcriptional co-expression signature as a conserved metabolic program shared across EBV-associated malignancies and reveals its immune cell-specific and dynamic characteristics. These findings provide new insight into the metabolic adaptation of EBV-associated malignancies and offer a conceptual framework for the development of metabolism-targeted therapeutic strategies for virus-associated cancers.

## 1 Introduction

Epstein-Barr virus (EBV) is the first identified human oncogenic virus and infects more than 95% of the adult population worldwide (Lee et al., 2025). Although most infected individuals remain asymptomatic throughout life with latent infection, persistent EBV infection is closely associated with multiple malignancies, including nasopharyngeal carcinoma, EBV-associated gastric cancer, extranodal NK/T-cell lymphoma, and several B-cell lymphomas (Young et al., 2016; Muckian et al., 2026). These malignancies arise from different tissues and exhibit substantial histological and molecular heterogeneity, yet they are all driven by persistent EBV infection. Despite recent advances in therapeutic strategies, patients with advanced or recurrent EBV-associated malignancies still face poor clinical outcomes, limited treatment options, and a lack of effective precision therapies (Wong et al., 2022; Sato et al., 2026). Therefore, systematically identifying the shared molecular basis of EBV-associated malignancies, particularly their conserved metabolic programs, is essential for understanding the common mechanisms underlying EBV-driven tumorigenesis and for discovering novel therapeutic targets.

Accumulating evidence indicates that metabolic reprogramming is a fundamental biological process by which oncogenic viruses promote malignant transformation. It also provides the metabolic basis for sustained tumor cell proliferation, immune evasion, and adaptation to the tumor microenvironment (Leung and Wang, 2023; Lee et al., 2025; Yee and Wang, 2025). EBV remodels multiple metabolic pathways, including glycolysis, lipid metabolism, nucleotide metabolism, one-carbon metabolism, and amino acid metabolism, through viral proteins such as latent membrane protein 1 (LMP1) and Epstein–Barr nuclear antigens (EBNAs), as well as host signaling pathways including PI3K/AKT, MYC, mTOR, and HIF-1 (Zhang et al., 2017; Burton and Gewurz, 2022; Yee and Wang, 2025). These metabolic alterations provide essential metabolic support for the survival, proliferation, and malignant transformation of infected cells. However, current studies on EBV-induced metabolic reprogramming have mainly focused on individual tumor types or specific metabolic pathways. Whether distinct EBV-associated malignancies share conserved metabolic programs remains largely unexplored. Moreover, conventional bulk transcriptomic analysis reflects the average gene expression of whole tissues and cannot resolve the cellular origin or cellular states underlying metabolic alterations. Consequently, the cellular basis of these conserved metabolic programs remains unclear.

In the present study, we integrated multiple public transcriptomic datasets from EBV-associated malignancies to systematically identify conserved metabolic programs shared across different tumor types through consensus differential gene analysis, single-gene metabolic pathway enrichment, and external validation. We further integrated single-cell transcriptomic data from extranodal NK/T-cell lymphoma and performed Scissor analysis, single-cell metabolic analysis, cell-cell communication analysis, and trajectory analysis to characterize the cellular distribution, metabolic states, and state-transition dynamics associated with the identified metabolic program across immune cell populations. Our study identified MTHFD1L-associated branched-chain amino acid (BCAA) degradation as a conserved metabolic program shared by EBV-associated malignancies and further characterized its cellular localization and dynamic features. These findings provide new insights into the metabolic reprogramming of EBV-associated malignancies and offer a theoretical basis for developing metabolism-targeted therapeutic strategies.

## 2 Materials and methods

The overall study design and main analytical workflow are shown in **Figure 1**.

### 2.1 Bulk transcriptome datasets and preprocessing

Four publicly available transcriptomic datasets of EBV-associated malignancies were obtained from the Gene Expression Omnibus (GEO) database. For each dataset, processed expression matrices containing normalized expression values were downloaded and used for subsequent bulk transcriptomic analyses. GSE12452 included nasopharyngeal carcinoma tissues and normal nasopharyngeal tissues. GSE51575 included EBV-positive gastric cancer tissues and control gastric tissues. GSE102203 included EBV-positive and EBV-negative plasmablastic lymphoma samples. GSE90579 was an NK/T-cell lymphoma (NKTL) transcriptomic dataset and was used for external validation. All expression matrices were processed using R software. Raw expression data were subjected to background correction, normalization, and log_2_ transformation. Differential expression analysis was performed using the limma package. Differentially expressed genes (DEGs) were identified using the thresholds of *P* < 0.05 and |log_2_FC| > 1. After DEGs were obtained from each dataset, the upregulated and downregulated genes were intersected separately according to the direction of expression change. The resulting genes were defined as consensus DEGs shared across EBV-associated malignancies.

### 2.2 Single-gene GSEA analysis

To systematically evaluate the associations between consensus DEGs and metabolic programs, single-gene gene set enrichment analysis (GSEA) was performed for each consensus DEG in GSE12452, GSE51575, and GSE102203. For each target gene, Pearson correlation coefficients were calculated between its expression level and the expression levels of all other genes in each dataset. Genes were then ranked in descending order according to their correlation coefficients to generate a ranked gene list. Enrichment analysis was performed using the GSEA function in the clusterProfiler package. Metabolic gene sets were obtained from the Kyoto Encyclopedia of Genes and Genomes (KEGG) database, and a total of 83 metabolism-related pathways were included. Statistical significance was defined as *P* < 0.05. Gene-metabolic pathway pairs were identified separately in each dataset, and conserved metabolic programs shared across datasets were subsequently screened.

### 2.3 Acquisition of KEGG metabolic genes

KEGG metabolism-related genes and pathway information were obtained through the KEGG REST API. All human metabolic pathways in the KEGG database were first retrieved, and the genes annotated to these pathways were extracted. After duplicate genes were removed, a total of 2,203 KEGG metabolism-related genes were obtained. This gene set was used for metabolic pathway annotation and gene–metabolic pathway association analysis.

### 2.4 Single-cell RNA sequencing analysis

The single-cell RNA sequencing (scRNA-seq) dataset GSE203663 was downloaded from the GEO database. This dataset was generated using the Illumina HiSeq X Ten platform and contains paired tumor tissue from 10 patients with extranodal NKTL. Single-cell data processing was performed using the Seurat package. Low-quality cells with fewer than 100 detected genes or ≥25% mitochondrial gene expression were excluded. Genes expressed in fewer than five cells were also removed. The expression matrix was normalized using the LogNormalize method, and 1,500 highly variable genes were identified for downstream analysis. Data were then scaled, followed by principal component analysis (PCA) for dimensionality reduction. Batch effects among samples were corrected using the Harmony algorithm. A shared nearest neighbor (SNN) graph was constructed based on the first 15 principal components, and cell clustering was performed accordingly. Cell distributions were visualized using t-distributed stochastic neighbor embedding (t-SNE). Cell types were manually annotated according to cluster-specific marker genes together with canonical cell markers (e.g., CD8A/CD8B for CD8+ T cells, CD4/IL7R for CD4+ T cells, NKG7/KLRD1/GNLY for NK cells, TRDC/TRGC for γδ T cells, MS4A1/CD19 for B cells, SDC1/MZB1 for plasma cells, CD14/LYZ/FCN1 for monocytes, CLEC4C/LILRA4 for pDCs, and CDH5/PECAM1/EMCN for endothelial cells). The default k-NN parameter (k = 20) and a clustering resolution of 0.1 were used.

Samples in the NKTL bulk transcriptomic dataset GSE90579 were divided into MTHFD1L-high and MTHFD1L-low groups according to the median expression level of MTHFD1L. The resulting phenotype labels were used as the bulk phenotype for Scissor analysis to identify single-cell populations associated with high MTHFD1L expression, which were defined as Scissor^+^ cells.

Metabolic pathway activities at the single-cell level were evaluated using the scMetabolism package. Differences in metabolic pathway activity between Scissor^+^ cells and all other cells were compared to characterize the activity patterns of the MTHFD1L-associated conserved metabolic program across different cell populations. Cell-cell communication analysis was performed using the CellChat package based on ligand-receptor interactions curated in the CellChatDB.human database to infer potential intercellular communication networks. Pseudotime analysis was conducted using the Slingshot package to infer the potential state-transition trajectories of different cell populations. Trajectories were inferred from t-SNE embeddings using Seurat cluster labels as input; because no *a priori* root population was known, no start cluster was manually specified and Slingshot inferred lineage roots and trajectories automatically (set.seed(123)). Finally, computational virtual knockout of MTHFD1L was performed using the scTenifoldKnk package. scTenifoldKnk was applied directly to count matrices restricted to the top 5,000 variable genes plus MTHFD1L; therefore cell k-NN/PC embedding parameters are not part of this method. The QC/network settings were qc_mtThreshold = 0.25, qc_minLSize = 100, nc_nNet = 20, and nc_nCells = 500. Candidate differentially regulated genes affected by MTHFD1L perturbation were identified by comparing gene regulatory networks before and after virtual knockout.

### 2.5 Statistical analysis

Statistical analyses were performed in R (version 4.3.1). Differential expression analysis, gene set enrichment analysis, and single-cell transcriptomic analyses were conducted using the corresponding R packages described above. All statistical tests were two-sided, and *P* < 0.05 was considered statistically significant.

## 3 Results

### 3.1 Identification of conserved metabolic programs shared across EBV-associated malignancies

To identify molecular features consistently shared across EBV-associated malignancies, differential expression analysis was performed in nasopharyngeal carcinoma (GSE12452), EBV-associated gastric cancer (GSE51575), and EBV-associated plasmablastic lymphoma (GSE102203). Distinct differential expression profiles were observed in all three datasets. Heatmaps showing the top 25 upregulated and top 25 downregulated genes in each dataset are presented in **Figures 1A–C**. Intersection analysis of the differentially expressed genes identified 103 consensus DEGs with consistent expression patterns across the three datasets, including 43 commonly upregulated genes and 60 commonly downregulated genes (**Figures 1D, E**).

**Figure 1:**
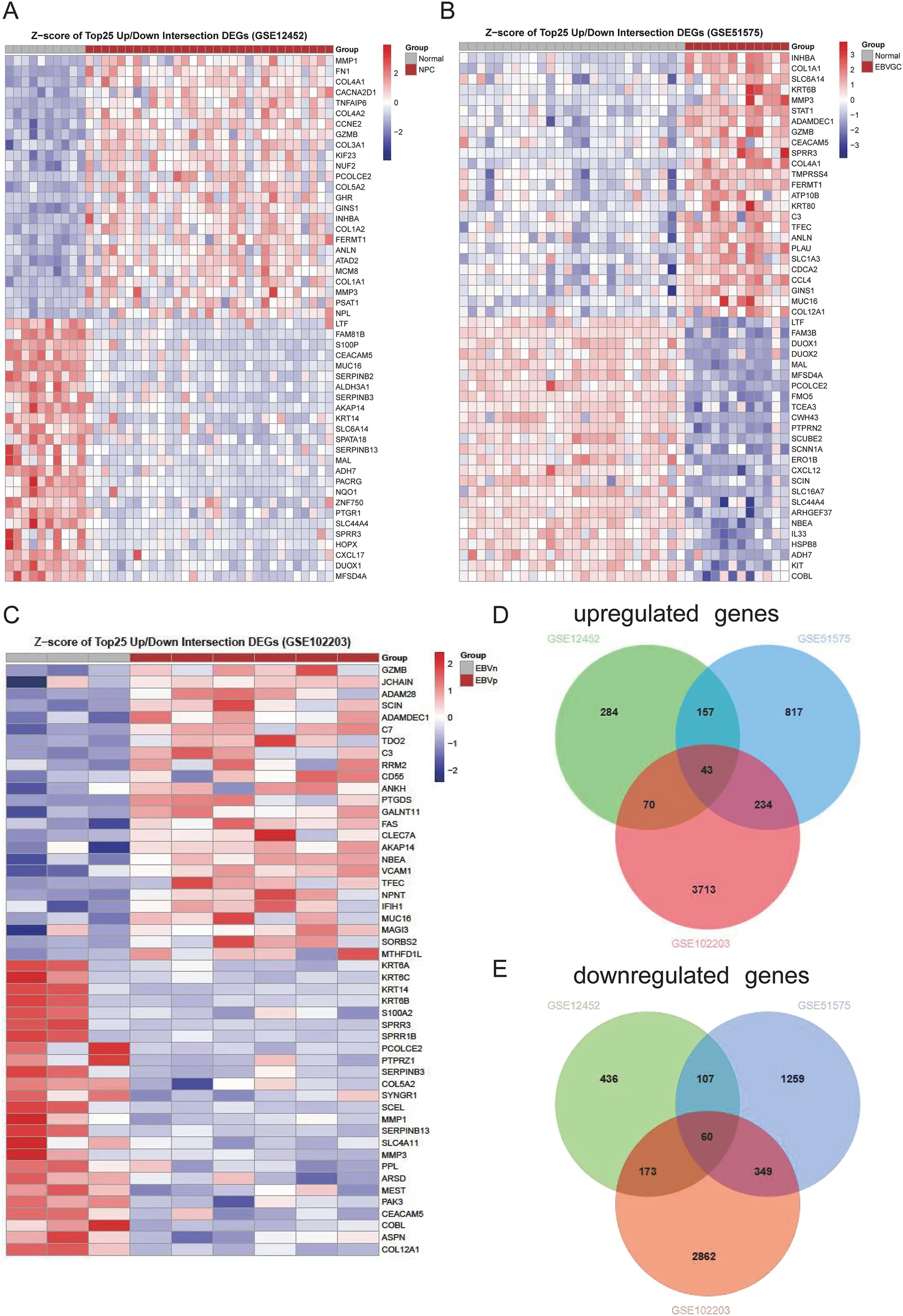
Identification of consensus differentially expressed genes across EBV-associated malignancies. **(A–C)** Heatmaps showing the top 25 upregulated and top 25 downregulated differentially expressed genes in the GSE12452, GSE51575, and GSE102203 datasets. Venn diagram showing the overlap of commonly upregulated **(D)** and downregulated **(E)** differentially expressed genes among the three datasets.

To further characterize the metabolic features represented by these consensus DEGs, single-gene GSEA was performed for each of the 103 consensus DEGs in the three datasets using 83 KEGG metabolic pathways as the reference gene sets. Gene-metabolic pathway pairs were subsequently constructed (**Supplementary Table 1**–**3**). A large number of significant gene–metabolic pathway pairs were identified in each dataset. Intersection analysis yielded 72 conserved significant gene–metabolic pathway pairs shared across all three datasets (**Figure 2A**), involving 25 genes (**Supplementary Table 4**).

**Figure 2:**
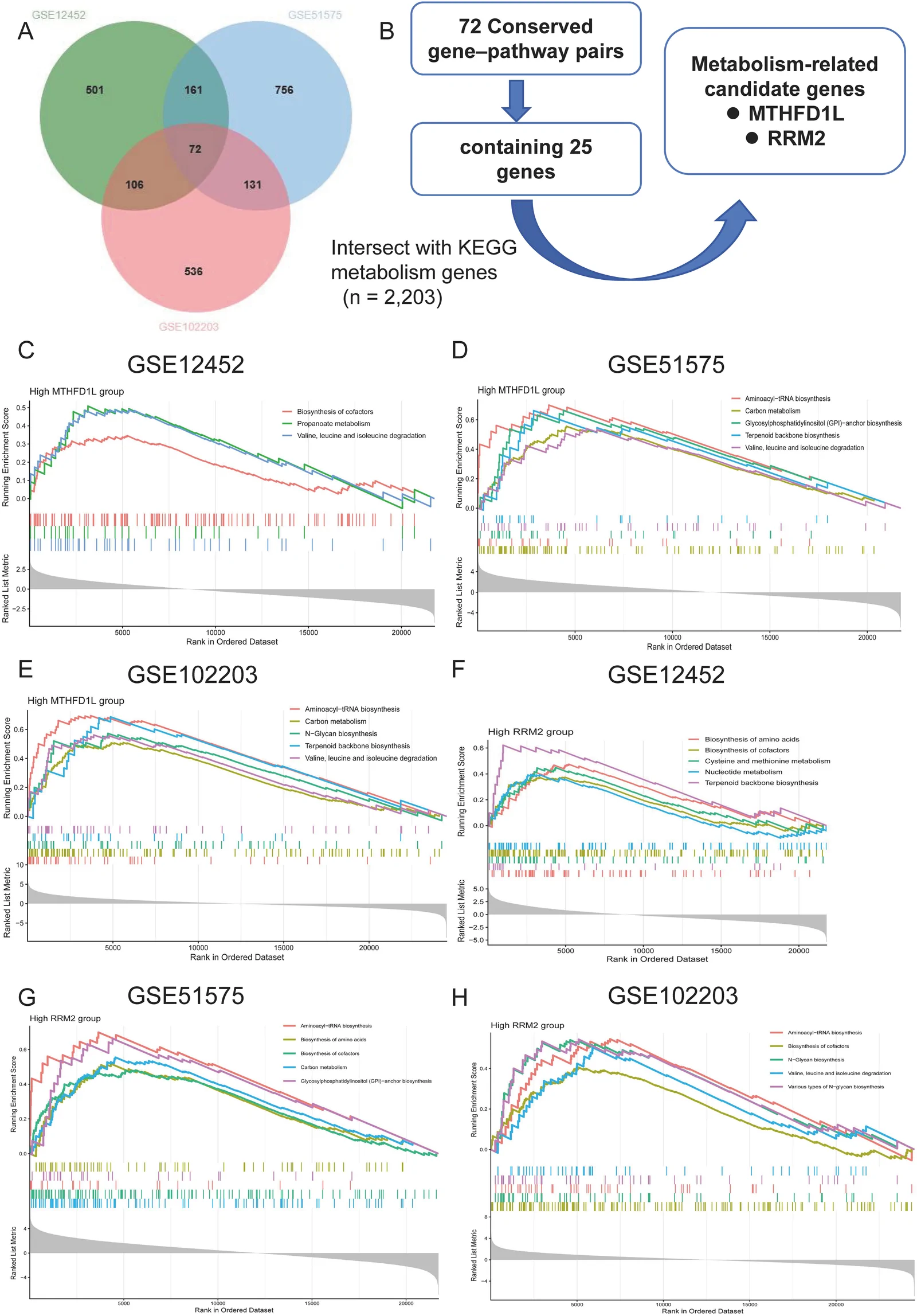
Identification of conserved metabolism-associated gene-pathway programs across EBV-associated malignancies. **(A)** Intersection analysis of significant gene-pathway pairs identified in the three datasets. **(B)** Workflow for identifying metabolism-related candidate genes. **(C–H)** Top five significantly enriched metabolic pathways associated with MTHFD1L and RRM2 in each of the three datasets, ranked by statistical significance.

As this study focused on metabolic reprogramming, the 25 genes were further intersected with 2,203 metabolism-related genes curated from the KEGG database. Only two metabolism-related genes, MTHFD1L and RRM2, were retained (**Figure 2B**; **Supplementary Table 5**). Further comparison showed that only three gene-metabolic pathway pairs exhibited consistent enrichment direction and statistical significance across all three datasets, including MTHFD1L—Biosynthesis of cofactors, MTHFD1L—Valine, leucine and isoleucine degradation, and RRM2—Biosynthesis of cofactors. Among them, MTHFD1L—Valine, leucine and isoleucine degradation showed consistently strong enrichment in all three datasets, with normalized enrichment scores (NESs) of 2.18, 2.01, and 2.02, respectively. The top five significantly enriched pathways associated with MTHFD1L and RRM2 in each dataset are shown in **Figures 2C–H**.

### 3.2 External validation of the MTHFD1L-associated BCAA degradation program

To validate the robustness of the identified metabolic program, an independent NK/T-cell lymphoma transcriptomic dataset (GSE90579) was analyzed. Single-gene GSEA was performed using MTHFD1L and RRM2 as the target genes. MTHFD1L remained significantly enriched in the Valine, leucine and isoleucine degradation pathway (NES = 1.93). In contrast, neither MTHFD1L—Biosynthesis of cofactors nor the RRM2-associated metabolic pathways reached statistical significance. The GSEA results for MTHFD1L and RRM2 in GSE90579 are presented in **Figures 3A–D**. These findings indicate that, among the identified metabolic programs, only the MTHFD1L-associated BCAA degradation program showed consistent enrichment across independent cohorts of different EBV-associated malignancies, further supporting its role as a conserved metabolic program shared by EBV-associated malignancies.

**Figure 3:**
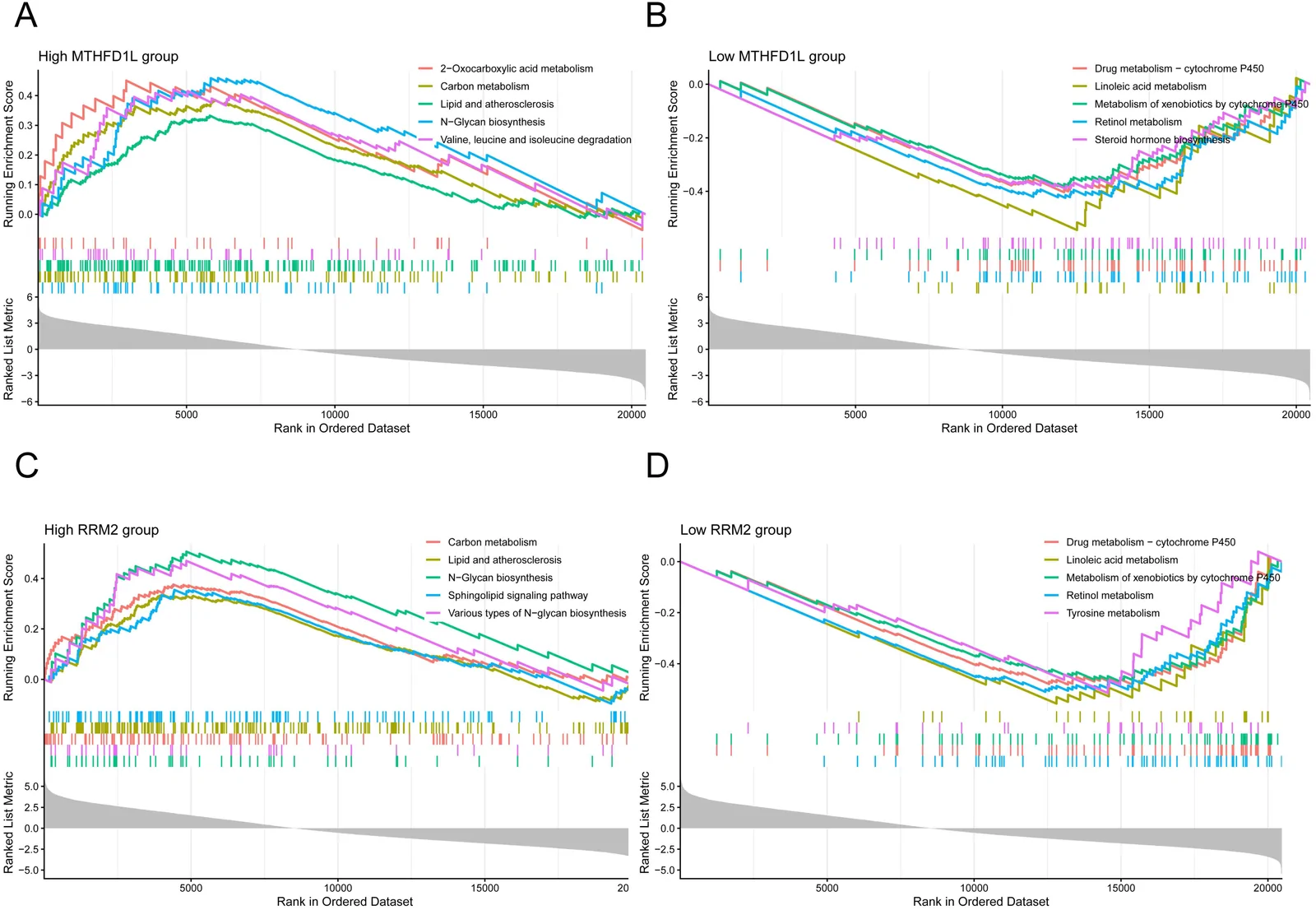
External validation of conserved metabolism-related pathways associated with candidate genes. Single-gene GSEA of MTHFD1L **(A, B)** and RRM2 **(C, D)** in the independent GSE90579 dataset.

### 3.3 Single-cell transcriptomic analysis reveals the cellular composition of the MTHFD1L-associated conserved metabolic program

To characterize the cellular distribution of the conserved metabolic program, we further analyzed the single-cell RNA sequencing dataset GSE203663 from NKTL. Following quality control, normalization, dimensionality reduction, clustering, and cell annotation, a total of 11 major cell types were identified, generating a single-cell transcriptomic atlas of NK/T-cell lymphoma (**Figures 4A–E**; **Supplementary Figure S2A-C**).

**Figure 4:**
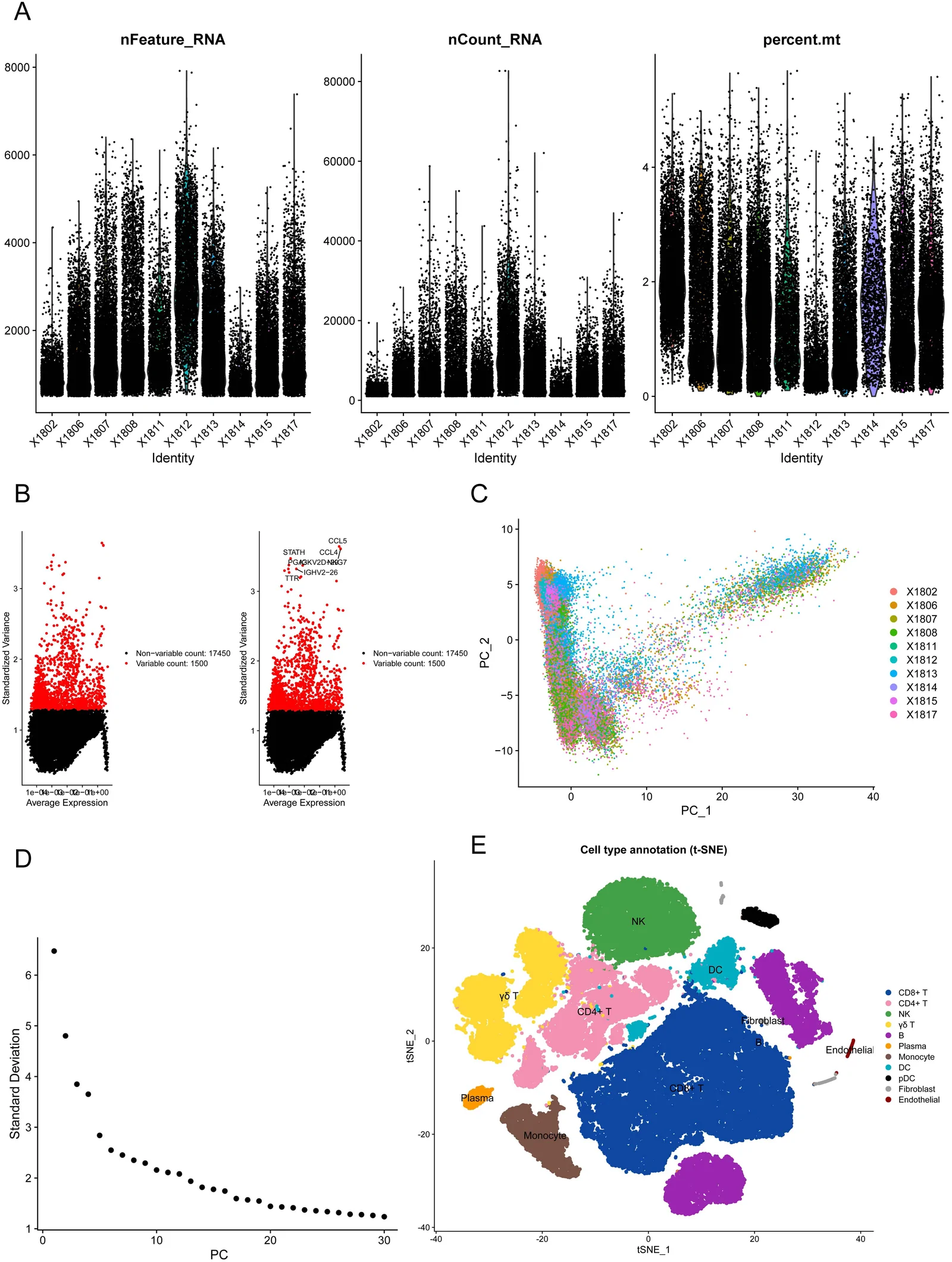
Preprocessing, dimensionality reduction, and cell-type annotation of the GSE203663 single-cell transcriptomic dataset. **(A)** Quality control violin plots for the ten samples, showing the number of detected genes (nFeature_RNA), total UMI counts (nCount_RNA), and the percentage of mitochondrial genes (percent.mt). **(B)** Identification of highly variable genes, with representative genes labeled. **(C)** PCA plot colored by sample. **(D)** Elbow plot used to determine the number of principal components for downstream analyses. **(E)** t-SNE visualization of annotated cell populations based on canonical marker genes.

Using the median MTHFD1L expression in GSE90579 as the bulk phenotype, the Scissor algorithm was applied to identify single-cell populations associated with the MTHFD1L-high phenotype (**Figure 5A**). Scissor^+^ cells were broadly distributed across multiple cell populations and were particularly enriched in CD4^+^ T cells, γδ T cells, and NK cells. Quantitative analysis further showed that these three cell types contained the largest numbers of Scissor^+^ cells (**Figure 5B**). In addition, they ranked among the highest in the proportion of Scissor^+^ cells within each corresponding cell type (**Supplementary Figure S3**), suggesting that they constitute the major cellular basis of the MTHFD1L-high phenotype.

**Figure 5:**
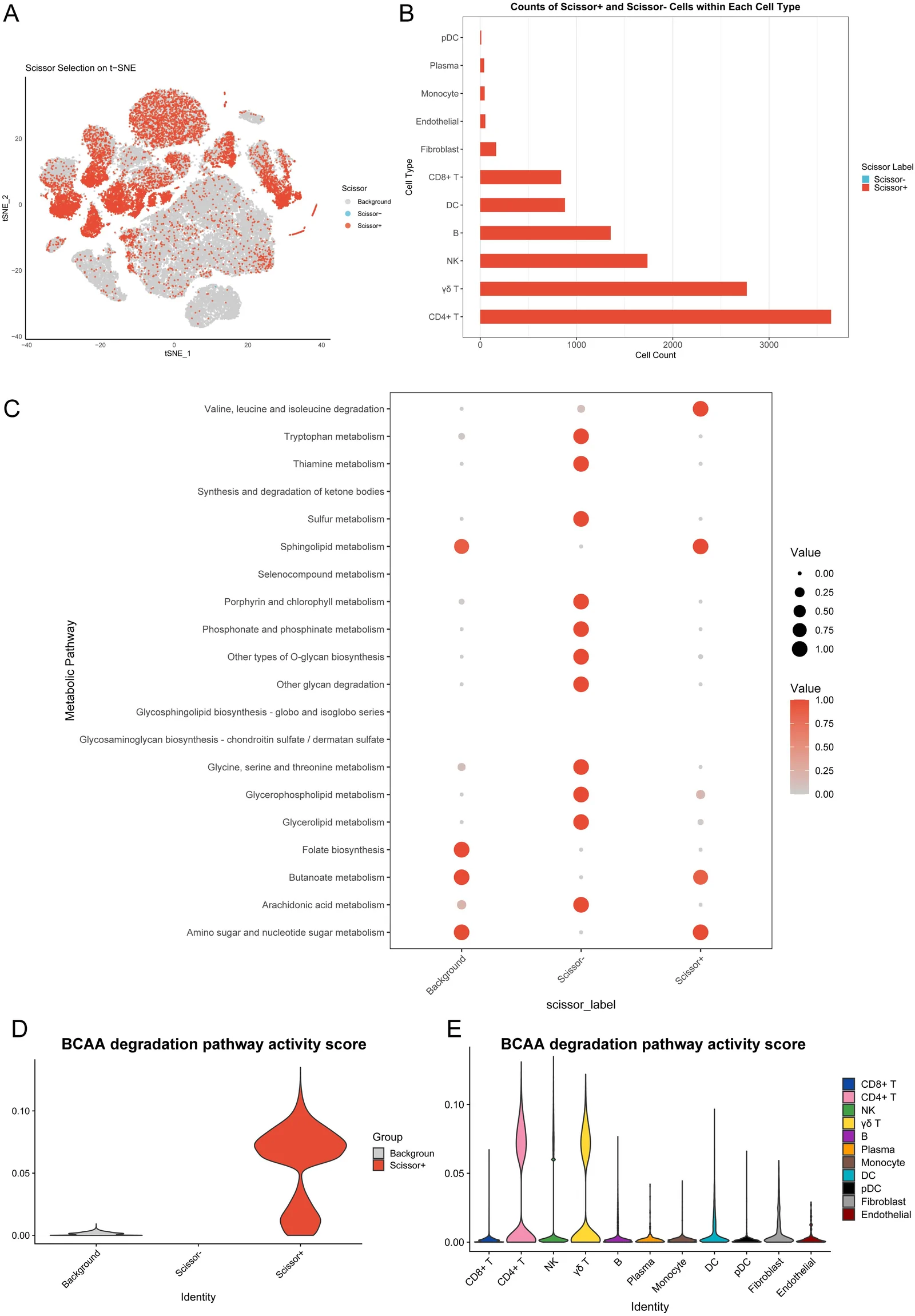
Identification and metabolic characterization of Scissor^+^ cells associated with the MTHFD1L-high phenotype. **(A)** Distribution of Scissor^+^, Scissor^−^, and background cells identified by Scissor analysis. **(B)** Number of Scissor^+^ cells across different cell types. **(C)** Bubble plot showing metabolic pathway activities in Scissor^+^ cells. **(D)** Comparison of branched-chain amino acid degradation pathway activity among Scissor^+^, Scissor^−^, and background cells. **(E)** Branched-chain amino acid degradation pathway activity across different cell types.

To further characterize the metabolic features of Scissor^+^ cells, metabolic pathway activities were evaluated using scMetabolism. Compared with all other cells, Scissor^+^ cells exhibited significantly higher activity of the Valine, leucine and isoleucine degradation pathway (**Figures 5C, D**). Cell type-specific analysis further demonstrated that this pathway showed the highest activity in CD4^+^ T cells, γδ T cells, and NK cells (**Figure 5E**). These results indicate that the MTHFD1L-associated BCAA degradation program is predominantly enriched in these immune cell subsets.

### 3.4 The MTHFD1L-associated metabolic program is accompanied by immune cell state remodeling

Because Scissor^+^ cells were predominantly enriched in CD4^+^ T cells, γδ T cells, and NK cells, we next investigated changes in cell–cell communication. CellChat analysis revealed substantial alterations in the overall communication patterns between these Scissor^+^ immune cell populations and surrounding cells. Scissor^+^ CD4^+^ T cells exhibited markedly reduced interactions with most other cell populations (**Figure 6A**). Similarly, Scissor^+^ γδ T cells and NK cells displayed weaker intercellular communication networks (**Figures 6B, C**), suggesting that the MTHFD1L-high phenotype is associated with remodeling of cell–cell communication.

**Figure 6:**
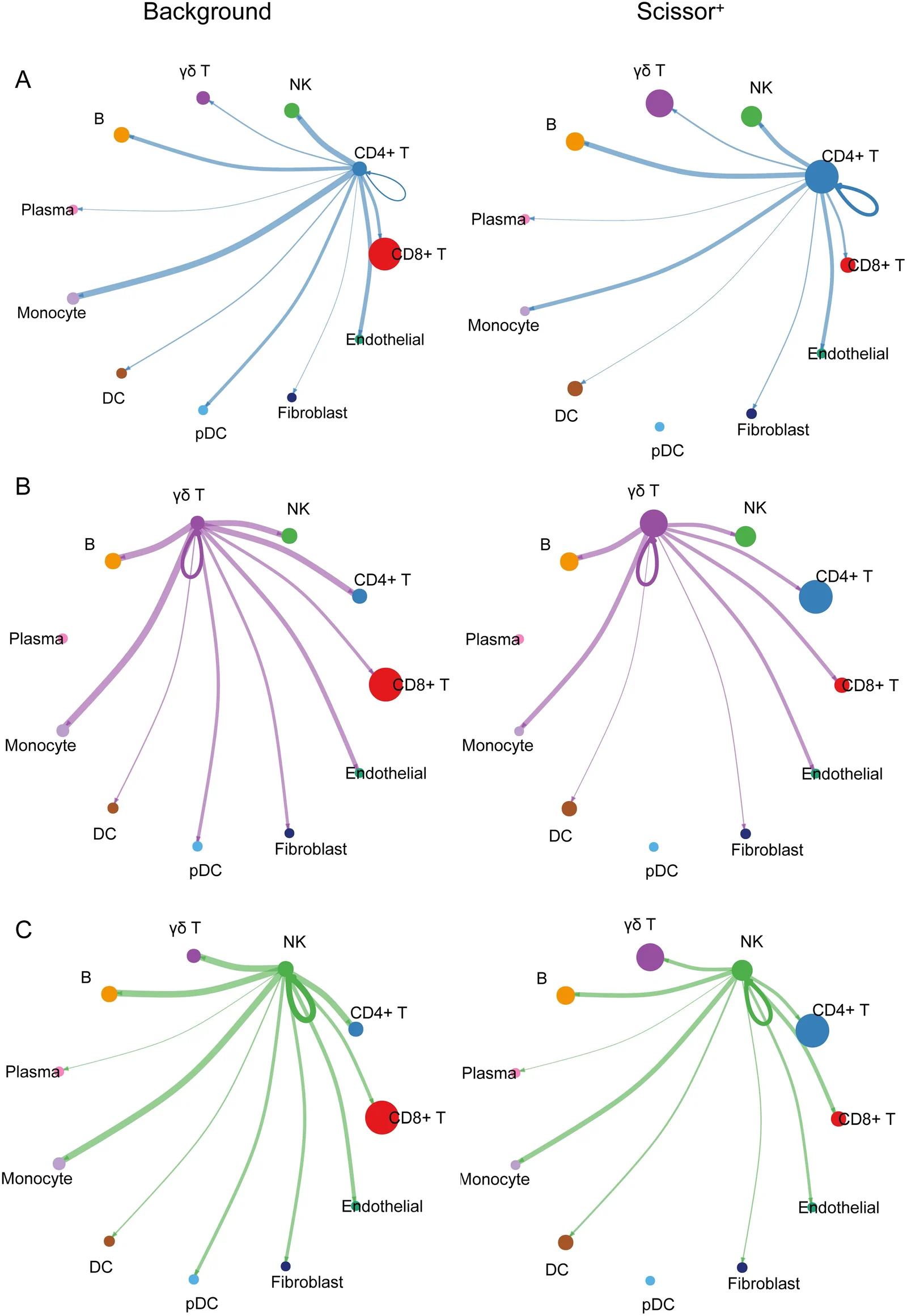
Cell–cell communication analysis between Scissor+ and background cells. **(A–C)** Comparison of the communication strength between CD4^+^ T cells **(A)**, γδ T cells **(B)**, or NK cells **(C)** and other cell populations in the background and Scissor^+^ groups.

Pseudotime trajectories were subsequently reconstructed for CD4^+^ T cells, γδ T cells, and NK cells. In CD4^+^ T cells, MTHFD1L expression gradually increased along the inferred state-transition trajectory (**Figures 7A–D**). In contrast, the activity of the Valine, leucine and isoleucine degradation pathway reached its highest level at the early cell state and progressively declined thereafter (**Figures 7E, F**). A similar dynamic pattern was observed in γδ T cells (**Supplementary Figure S4**). By comparison, neither MTHFD1L expression nor the activity of the associated metabolic pathway changed substantially during pseudotime in NK cells (**Supplementary Figure S5**). These findings suggest that the MTHFD1L-associated BCAA degradation program exhibits cell type-dependent and dynamic state-specific characteristics, and that metabolic activity is not always synchronized with MTHFD1L expression.

**Figure 7:**
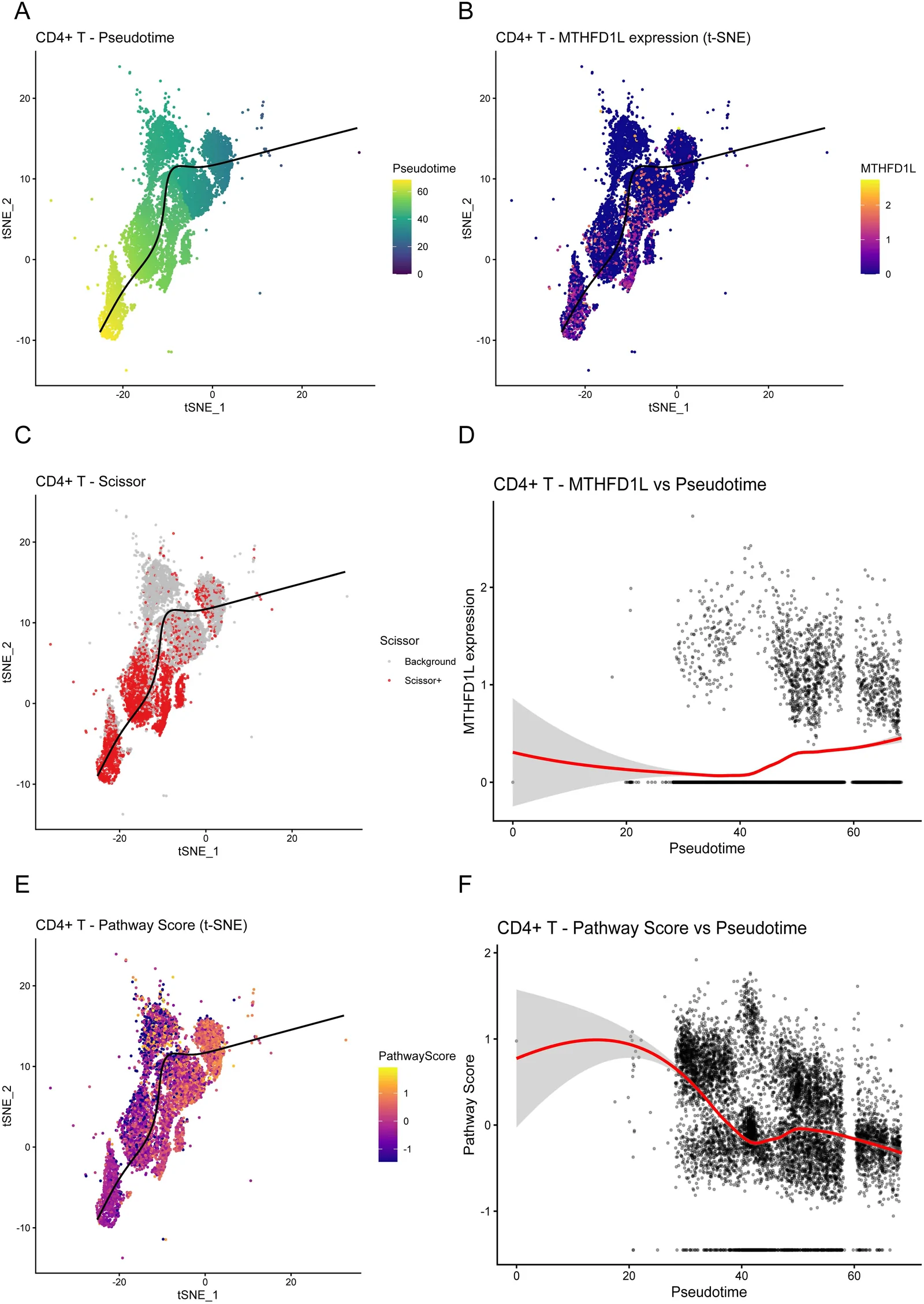
Dynamic changes in MTHFD1L expression and metabolic pathway activity along the CD4^+^ T-cell pseudotime trajectory. **(A)** t-SNE plot of CD4^+^ T cells colored by pseudotime, with the black curve indicating the inferred differentiation trajectory. **(B)** t-SNE plot showing MTHFD1L expression in CD4^+^ T cells. **(C)** Distribution of Scissor^+^ cells along the CD4^+^ T-cell pseudotime trajectory. Red indicates Scissor^+^ cells, gray indicates background cells, and the black curve represents the inferred differentiation trajectory. **(D)** Dynamic changes in MTHFD1L expression along pseudotime. The red line represents the smoothed fitted curve, and the gray shaded area indicates the 95% confidence interval. **(E)** t-SNE plot of CD4^+^ T cells colored by metabolic pathway enrichment scores, with the black curve indicating the inferred differentiation trajectory. **(F)** Dynamic changes in metabolic pathway enrichment scores along pseudotime. The red line represents the smoothed fitted curve, and the gray shaded area indicates the 95% confidence interval.

### 3.5 Virtual knockout reveals distinct regulatory networks of MTHFD1L in different immune cell types

To further investigate the potential functions of MTHFD1L in different immune cell populations, virtual knockout analysis was performed in CD4^+^ T cells, γδ T cells, and NK cells using ScTenifoldKnk. Knockout of MTHFD1L induced varying degrees of perturbation in gene regulatory networks across all three cell types. However, the numbers of affected genes and the extent of network disruption differed substantially among cell types (**Figures 8A, C, E**; **Supplementary Table 6**). Ranking analysis of the perturbed genes further demonstrated that the key regulatory genes influenced by MTHFD1L were largely cell type-specific (**Figures 8B, D, F**). These results suggest that MTHFD1L may contribute to the maintenance of immune cell states and functional regulation through distinct cell type-dependent regulatory networks.

**Figure 8:**
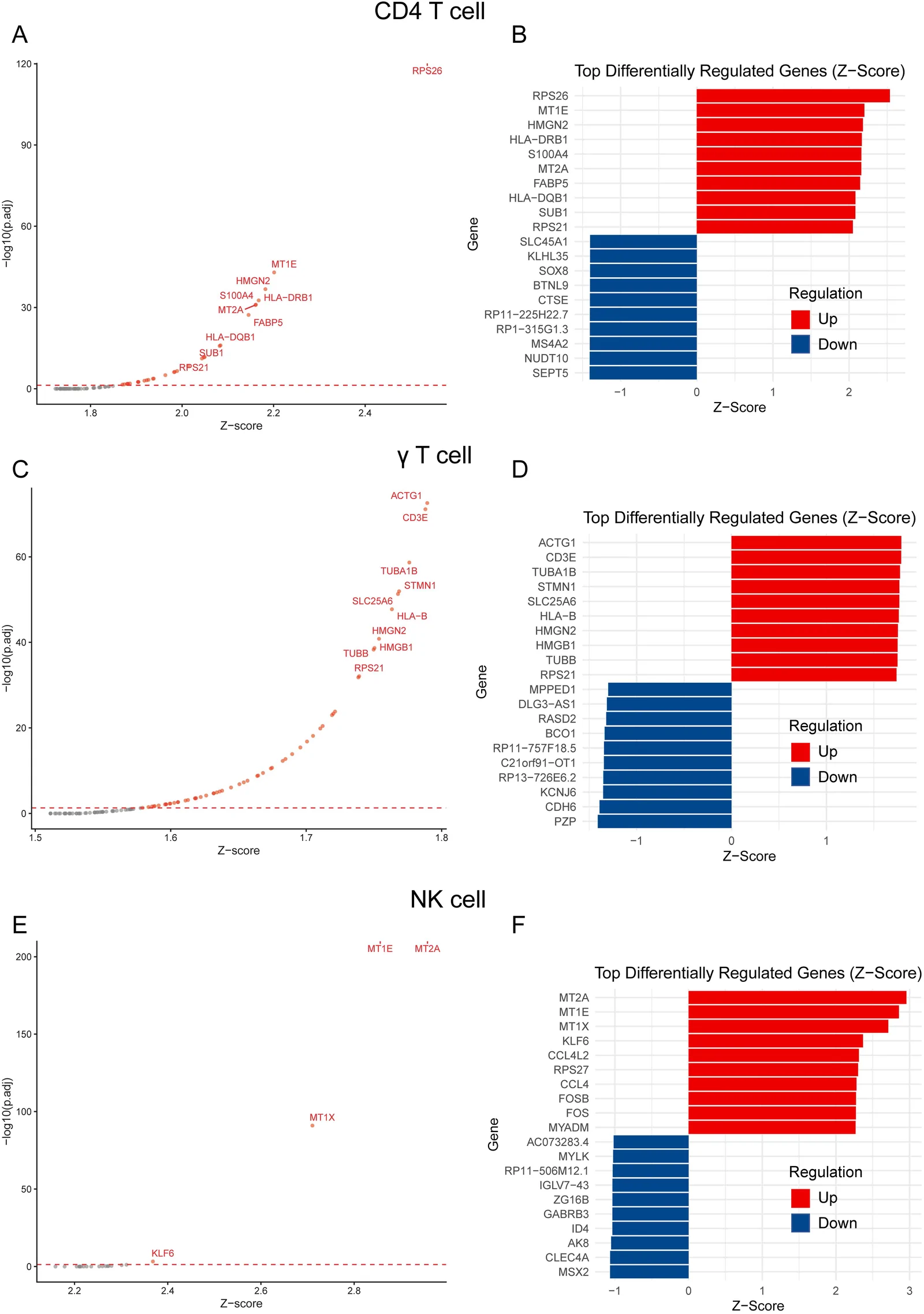
Predicted transcriptomic perturbations following in silico knockout of MTHFD1L. Differential expression analysis following virtual knockout of MTHFD1L in CD4^+^ T cells **(A, B)**, γδ T cells **(C, D)**, and NK cells **(E, F)**. **(A, C, E)** Volcano plots showing differentially expressed genes, with the top 10 significant genes labeled. **(B, D, F)** Ranked bar plots of differentially expressed genes based on Z-scores, highlighting the top upregulated and downregulated genes.

## 4 Discussion

In the present study, we identified an MTHFD1L-associated BCAA degradation program as a conserved metabolic feature shared across multiple EBV-associated malignancies. This metabolic program was consistently observed in three independent transcriptomic datasets and was further validated in an external cohort. Single-cell analysis further demonstrated that this program was predominantly enriched in CD4^+^ T cells, γδ T cells, and NK cells, exhibiting marked cell type- and state-dependent characteristics. These findings suggest that, despite their distinct tissue origins and biological characteristics, EBV-associated malignancies may converge on a common metabolic adaptation. Our study therefore provides a new perspective for understanding metabolic reprogramming in EBV-associated malignancies.

In recent years, a growing number of studies has found that virus-induced metabolic reprogramming extends beyond individual metabolic pathways and instead involves coordinated remodeling of interconnected metabolic networks (Bappy et al., 2024). Our findings further support this concept. Notably, the conserved metabolic program identified in this study was not the canonical glycolytic or fatty acid metabolic pathway but rather the BCAA degradation pathway. In recent years, BCAA metabolism has emerged as a central metabolic hub linking energy metabolism, redox homeostasis, and immune regulation. BCAA catabolism not only supplies intermediates for the tricarboxylic acid cycle but also contributes to tumor progression by regulating mTOR signaling, mitochondrial function, and immune cell effector states (Choi et al., 2024; Cai et al., 2025; Xiong et al., 2025). On the other hand, MTHFD1L is a key enzyme in the mitochondrial one-carbon metabolism pathway. It mediates the conversion and export of mitochondrial one-carbon units and provides metabolic support for nucleotide biosynthesis and cellular redox homeostasis (Ducker and Rabinowitz, 2017). Previous studies have shown that MTHFD1L is frequently upregulated in multiple human malignancies, where it promotes tumor cell proliferation and metabolic adaptation by maintaining NADPH levels, limiting oxidative stress, and supporting anabolic metabolism. Elevated MTHFD1L expression has also been associated with tumor progression and poor clinical outcomes (Lee et al., 2017). To date, however, there is no direct evidence that MTHFD1L regulates BCAA degradation. Nevertheless, a potential metabolic connection between these pathways is biologically plausible, particularly in immune cells, where mitochondrial metabolism is dynamically remodeled according to cellular activation and functional states. Mitochondrial one-carbon metabolism supports redox balance and biosynthetic demands, whereas BCAA catabolism contributes carbon substrates to the TCA cycle and is closely linked to nutrient-sensing pathways such as mTOR. Thus, these pathways may converge at the level of mitochondrial substrate utilization, redox homeostasis, and nutrient-responsive signaling rather than through direct enzymatic regulation. From this perspective, MTHFD1L may reflect a broader mitochondrial metabolic state associated with BCAA utilization rather than acting as a direct driver of BCAA degradation. The stable association between MTHFD1L and BCAA degradation across multiple independent cohorts therefore raises the possibility that mitochondrial one-carbon metabolism and BCAA catabolism are functionally coordinated within the metabolic network of EBV-associated malignancies. Further studies integrating stable isotope tracing, metabolic flux analysis, and functional manipulation of MTHFD1L will be required to determine the mechanistic basis of this potential metabolic crosstalk.

Pseudotime analysis further demonstrated that the MTHFD1L-associated conserved metabolic program exhibited pronounced state dependency rather than representing a static metabolic feature. As CD4^+^ T cells and γδ T cells progressed along the inferred trajectories, MTHFD1L expression gradually increased, whereas the activity of the BCAA degradation pathway progressively declined. Although this pattern was not fully consistent with the overall positive association observed in the bulk transcriptomic analysis, the two analyses reflect metabolic characteristics at different biological levels. Bulk transcriptomic analysis captures average metabolic features across patient samples, whereas single-cell pseudotime analysis enables the characterization of dynamic metabolic remodeling during continuous cell-state transitions (Ding et al., 2022; Golushko and Buzdin, 2026). Previous studies have demonstrated that immune cells undergo continuous metabolic remodeling during activation, effector differentiation, and exhaustion. Their metabolic phenotypes are regulated not only by the expression of metabolic genes but also by nutrient availability, mitochondrial function, metabolite signaling, and the tumor microenvironment (Leone and Powell, 2020; Chapman and Chi, 2024). Consequently, metabolic gene expression does not necessarily parallel actual metabolic flux. Our findings thus support state-dependent metabolic remodeling of immune cells during cell-state transitions, rather than a genuine biological contradiction between MTHFD1L expression and BCAA degradation activity.

Meanwhile, CellChat analysis revealed that Scissor^+^ immune cells did not simply exhibit increased or decreased communication activity but instead underwent global remodeling of intercellular communication patterns. Increasing evidence suggests that metabolic reprogramming not only fulfills the energetic and biosynthetic demands of tumor cells but also reshapes the tumor immune microenvironment by regulating metabolite signaling, cytokine and chemokine production, and immune checkpoint-related pathways, thereby altering communication between tumor and immune cells (Elia and Haigis, 2021; Kao et al., 2022). In light of these findings, we speculate that the MTHFD1L-associated conserved metabolic program may not only reflect a shared metabolic state across EBV-associated malignancies but also be closely associated with immune cell state transitions and remodeling of intercellular communication networks. This hypothesis warrants further investigation using spatial transcriptomics, metabolic flux analysis, and functional experiments.

Despite the robustness gained from integrating transcriptomic datasets across multiple cancer types with single-cell transcriptomic analysis, this study has several limitations. First, All analyses rely exclusively on retrospective public transcriptomic datasets; there is no in-house prospective patient cohort RNA-seq validation. Sample heterogeneity across independent public datasets (different sequencing platforms, EBV quantification methods, tissue processing protocols) may introduce batch confounding effects, even after computational normalization. Moreover, because no EBV-negative tumor or normal tissue was used as a comparator, the conserved program should be interpreted as shared among EBV-associated tumors rather than EBV-specific. Second, The conserved MTHFD1L-BCAA degradation signature is identified via correlational transcriptomics; the manuscript cannot confirm direct molecular regulatory interaction between MTHFD1L enzymatic activity and BCAA catabolic flux. Stable isotope metabolic tracing, CRISPR overexpression/knockout of MTHFD1L, and EBV tumor xenograft models are required to functionally validate this axis. Third, Spatial transcriptomics/metabolomics data is absent; the study cannot resolve spatial localization of MTHFD1L-high BCAA-active immune cells within tumor parenchyma versus stromal regions of the EBV-associated tumor microenvironment. Finally, No clinical outcome correlation analysis is performed: the manuscript does not link MTHFD1L-BCAA signature expression to patient survival, treatment response, or disease stage across EBV malignancies, limiting translational therapeutic relevance of the signature. In addition, Scissor+ enrichment may partly reflect bulk-level immune composition rather than cell-intrinsic metabolic states; our transcriptomic approach cannot fully separate compositional effects from cell-intrinsic properties, and sorted-cell or spatial transcriptomic studies will be required to resolve this limitation.

## 5 Conclusions

In conclusion, through integrated cross-cancer transcriptomic analysis and single-cell transcriptomic characterization, we systematically identified an MTHFD1L-associated conserved metabolic program shared across EBV-associated malignancies. We further demonstrated that this program is predominantly enriched in immune cells and exhibits dynamic, cell state-dependent metabolic characteristics. These findings expand our current understanding of metabolic reprogramming in EBV-associated malignancies and suggest that EBV-associated tumors arising from distinct tissue origins may share common metabolic adaptation mechanisms. Although the underlying regulatory mechanisms require further experimental validation, this conserved metabolic program provides a valuable framework for understanding the shared biology of EBV-associated malignancies. Importantly, the conserved transcriptomic association between MTHFD1L and BCAA degradation highlights this metabolic axis as a potential shared metabolic vulnerability and warrants further investigation as a therapeutic target across heterogeneous EBV-associated malignancies.

## Data Availability

The datasets presented in this study can be found in online repositories. The names of the repository/repositories and accession number(s) can be found in the article/**Supplementary Material**.

## Supplementary material

Supplementary Figure 1 The overall study design and main analytical workflow.

Supplementary Figure 2 Additional quality assessment and cell clustering of the GSE203663 single-cell transcriptomic dataset. (A) PCA loading plot showing the top genes contributing to the principal components. (B) t-SNE plot of unsupervised cell clusters before cell-type annotation. (C) Heatmap of representative marker genes across cell clusters.

Supplementary Figure 3 Proportion of Scissor+ cells across different cell types.

Supplementary Figure 4 Dynamic changes in MTHFD1L expression and metabolic pathway activity along the γδ T-cell pseudotime trajectory. (A) t-SNE plot of γδ T cells colored by pseudotime, with the black curve indicating the inferred differentiation trajectory. (B) t-SNE plot showing MTHFD1L expression in γδ T cells. (C) Distribution of Scissor+ cells along the γδ T-cell pseudotime trajectory. Red indicates Scissor+ cells, gray indicates background cells, and the black curve represents the inferred differentiation trajectory. (D) Dynamic changes in MTHFD1L expression along pseudotime. The red line represents the smoothed fitted curve, and the gray shaded area indicates the 95% confidence interval. (E) t-SNE plot of γδ T cells colored by metabolic pathway enrichment scores, with the black curve indicating the inferred differentiation trajectory. (F) Dynamic changes in metabolic pathway enrichment scores along pseudotime. The red line represents the smoothed fitted curve, and the gray shaded area indicates the 95% confidence interval.

Supplementary Figure 5 Dynamic changes in MTHFD1L expression and metabolic pathway activity along the NK-cell pseudotime trajectory. (A) t-SNE plot of NK cells colored by pseudotime, with the black curve indicating the inferred differentiation trajectory. (B) t-SNE plot showing MTHFD1L expression in NK cells. (C) Distribution of Scissor+ cells along the NK-cell pseudotime trajectory. Red indicates Scissor+ cells, gray indicates background cells, and the black curve represents the inferred differentiation trajectory. (D) Dynamic changes in MTHFD1L expression along pseudotime. The red line represents the smoothed fitted curve, and the gray shaded area indicates the 95% confidence interval. (E) t-SNE plot of NK cells colored by metabolic pathway enrichment scores, with the black curve indicating the inferred differentiation trajectory. (F) Dynamic changes in metabolic pathway enrichment scores along pseudotime. The red line represents the smoothed fitted curve, and the gray shaded area indicates the 95% confidence interval.
